# Supplementary material for: Systemic DNA Damage and Repair Activity Vary by Race in Breast Cancer Survivors
Source: Cancers (Basel). 2024 May 9;16(10):1807. doi: 10.3390/cancers16101807 (PMC11119753; doi:10.3390/cancers16101807)
Supplement: Supplementary file 1 [file cancers-16-01807-s001.zip › cancers-2977021-supplementary.pdf]

Cancers **2024**,

Supplementary materials

Systemic DNA Damage and Repair Activity Vary by Race in Breast Cancer Survivors

Shraddha Divekar, Ryan Kritzer, Haokai Shu, Keval Thakkar , Jennifer Hicks, Mary G. Mills, Kepher Makambi, Chiranjeev Dash * and Rabindra Roy *

Table S1. Demographics of the Breast Cancer survivors whose samples were utilized in this study at GLCCC

Characteristics, *N (%)*	All participants *(N=25)*	Non-Hispanic White *(N=12; 48%)*	Non-Hispanic Black *(N=13; 52%)*
Age[a]			
>40 year	24 (96)	11 (92)	13 (100)
(Mean±SD)	58.2±9.6	54.5±9.6	61.5±8.6
Tumor stage			
I	16 (64)	8 (67)	8 (62)
II	6 (24)	3 (25)	3 (23)
III	3 (12)	1 (8)	2 (15)
Histology			
Invasive	25 (100)	12 (100)	13 (100)
In situ	0	0	0
Body Mass Index *(Mean±SD)*	30.6±6.2	30.4±8.1	30.8±4.4
Smoking status			
Non-smokers	25 (100)	12 (100)	13 (100)
Smokers	0	0	0
Prior history of other cancers			
Yes	0	0	0
No	25 (100)	12 (100)	13 (100)
Surgery			
Yes	17 (68)	8 (67)	9 (69)
No	5 (32)	4 (33)	4 (31)
Radiation therapy			
Yes	8 (32)	3 (25)	5 (38)
No	17 (68)	10 (75)	10 (62)
Chemotherapy			
Yes	17 (68)	8 (67)	9 (69)
No	8 (32)	4 (33)	4 (31)

[a]Age at the time of the blood draw

Figure S1. Alkaline and neutral CometChip assay optimization for DNA damage, Bleomycin dose, and repair kinetics

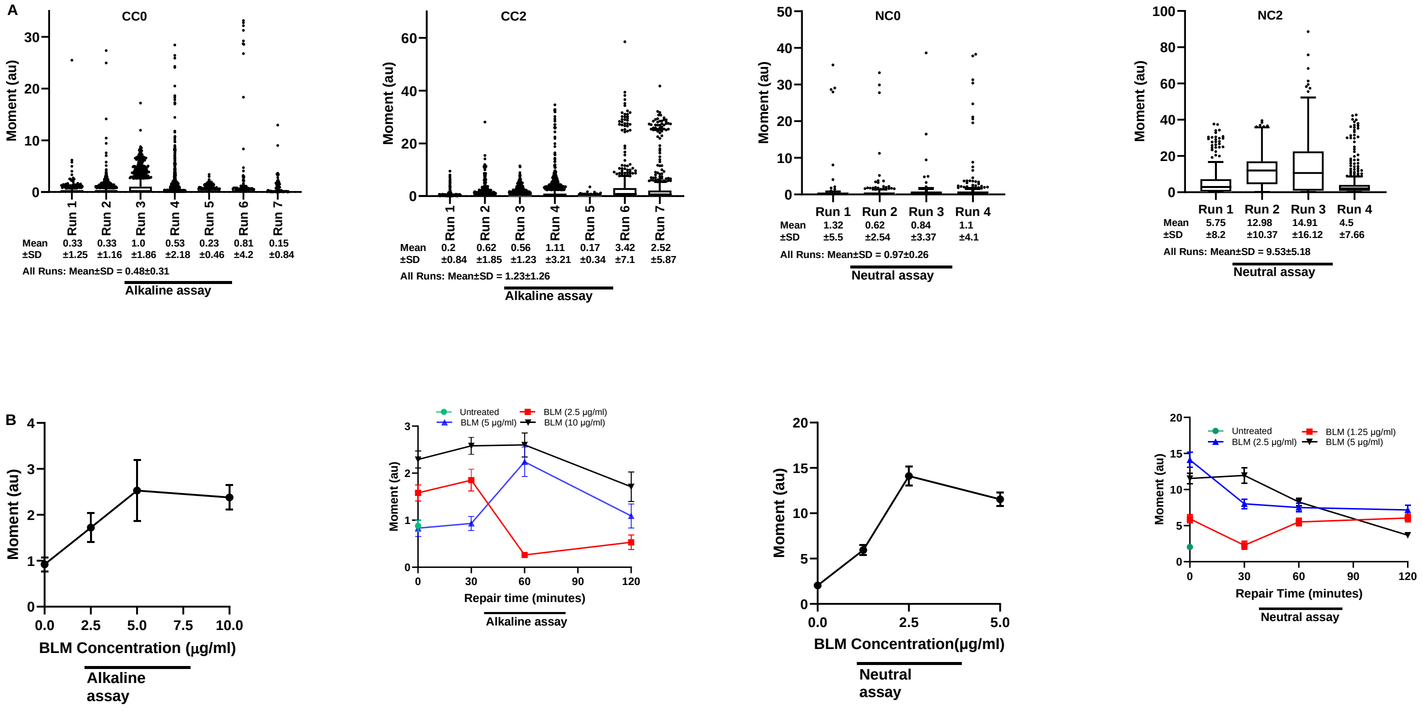

Figure S1: (A) Scatter plots of Trevigen reference Alkaline assay control (CC0) and damaged (CC2) cells and Neutral assay control (NC0) and damaged (NC2) cells on different days of experiments are shown with mean and standard deviation (SD) values. The mean and SD of all runs for control and damaged cells are also shown. (B) Alkaline and neutral CometChip assay optimization for DNA damage, Bleomycin dose, and repair kinetics. Dose-dependent response to different BLM concentrations (first and third graphs) and DNA Repair time kinetics (second and fourth graphs). The buffy coat cells from a non-cancer woman were treated with different concentrations of Bleomycin (BLM). The 0 also represents the basal damage in the buffy coat cells. The tail moment is shown for both BLM treatment and DNA Repair. Five hundred to one thousand single cells in buffy coats were analyzed in Alkaline and Neutral CometChip assays.

Figure S2: Effect of race on basal (A), damage susceptibility (B), and repair (C) of BLM-induced double-strand break in cancer-free Black (NHB) and White (NHW) leukocytes.

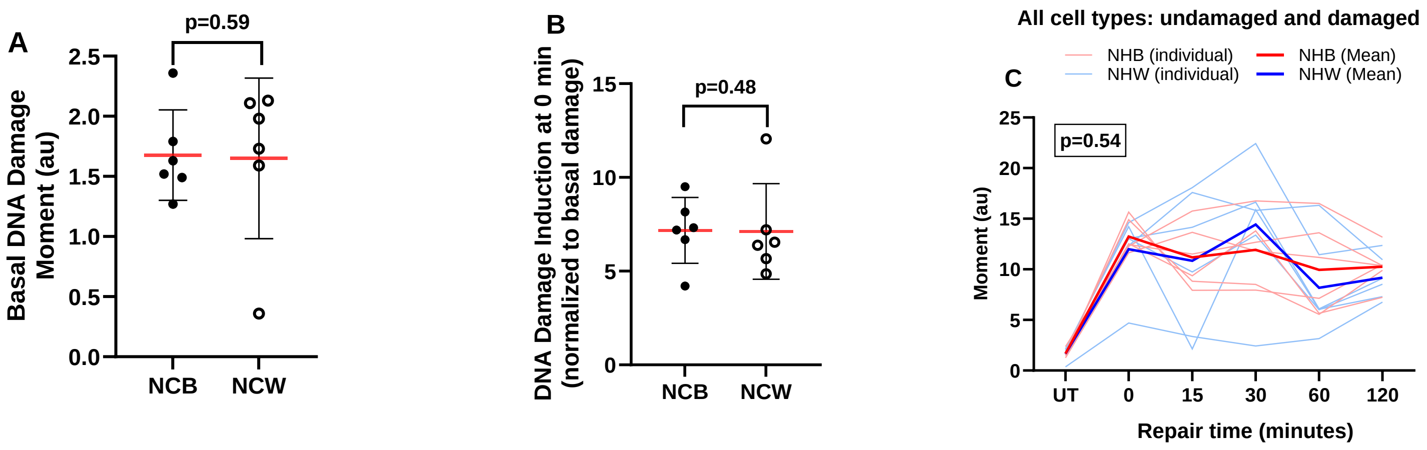

Figure S2: Effect of race on basal (A), and damage susceptibility (B) and repair (C) of BLM-induced double-strand break in cancer-free NHB and NHW leukocytes. Differences in DNA damage (Mean± SD) between NHB and NHW (A and B) were assessed using unpaired, 2-tail Mann-Whitney test. DNA damage induction in panel B was calculated using the formula: [DNA damage (Moment) after BLM treatment – Basal DNA damage (Moment) before BLM treatment]/Basal DNA damage (Moment) before BLM treatment. A repeated measures ANOVA was conducted to assess the impact of race on repair kinetics. p-value represents the statistical significance of the interaction of race with repair activity at different time points. $p<0.05$ is considered significant.
